# Poor Teeth the Cause of Many Ills

**Published:** 1883-06

**Authors:** 


### POOR TEETH THE CAUSE OF MANY ILLS.

It appears not to be generally understood, even among cultivated people, although the fact has been dwelt upon with emphasis by the best medical authorities, that the presence of carious, crowded, or asymmetrical teeth in the human mouth is the progenitor of a long train of nervous diseases, comprising not only facial neuralgia and its concomitant troubles, but diseases of the ear, inflammatory as well as functional, eventuating often in partial loss of hearing, defects of vision, naso-pharyngeal catarrh, and other tormenting maladies. One of our acutest and most successful specialists in the treatment of nervous diseases has become so fully convinced by long experience of the part played by defective teeth in the development, not of neuralgia only, but even of the more obscure neuroses, that he always insists, as a condition precedent to the acceptance of the case, that a thorough examination of the cavity of the mouth shall be undertaken by a competent dentist, for, he says, not only may a single diseased tooth result in persistent nervous disturbance, but diseases of the brain, decay or perversion of the mental faculties, even epilepsy and tetanic spasms often have their starting point in dental irritations; and he has observed cases in which, while laying the foundation for a long train of nervous troubles, the irritated organ itself gave no sign, either by local pain or vague discomfort, of the agency it was constantly exerting to produce serious disturbance at some distant point.

